# Traditional Chinese herbal medicines suppress endometriosis development through modulating macrophage-mediated immune responses in the peritoneal cavity

**DOI:** 10.37796/2211-8039.1706

**Published:** 2026-06-01

**Authors:** Cherry Yin-Yi Chang, Kung-Pin Chang, Pei-Fen Lee, Hsiang-Hao Chuang, Yun-Yi Su, Yu-Chi Chang, Man-Ju Yan, Chih-Mei Chen, Tritium Hwang, Huan-Yuan Chen, Chia-Jung Li, Zhi-Hong Wen, Kuan-Hao Tsui, Lun-Chien Lo, Jim Jinn-Chyuan Sheu

**Affiliations:** a Department of Obstetrics and Gynecology, China Medical University Hospital, Taichung, Taiwan; b School of Medicine, China Medical University, Taichung, Taiwan; c Institute of Biomedical Sciences, National Sun Yat-sen University, Kaohsiung, Taiwan; d Department of Emergency, Kaohsiung Municipal Ta-Tung Hospital, Kaohsiung, Taiwan; e Genetics Center, China Medical University Hospital, Taichung, Taiwan; f Institute of Biomedical Sciences, Academia Sinica, Taipei, Taiwan; g Department of Obstetrics and Gynecology, Kaohsiung Veterans General Hospital, Kaohsiung, Taiwan; h Institute of Biopharmaceutical Sciences, National Sun Yatsen University, Kaohsiung, Taiwan; i Department of Marine Biotechnology and Resources, National Sun Yat-sen University, Kaohsiung, Taiwan; j School of Chinese Medicine, China Medical University, Taichung, Taiwan; k Institute of Precision Medicine, National Sun Yat-sen University, Kaohsiung, Taiwan; l Department of Biotechnology, Kaohsiung Medical University, Kaohsiung, Taiwan

**Keywords:** Endometriosis, Traditional Chinese herbal medicine (TCHM), Macrophage, Danggui buxue tang (DBT), Shaofu zhuyu tang (SZT)

## Abstract

**Background:**

Due to unique dietary culture, Asian patients with endometriosis usually seek assistance through traditional Chinese herbal medicine (TCHM), making combined treatments of Western and Chinese medicine quite common in Asia. However, limited molecular evidence has been provided to demonstrate the therapeutic benefits of TCHM in treating endometriosis.

**Aims:**

To investigate the functional impacts of TCHM treatment in their traditional formulae on peritoneal immunity, lesion growth, and adhesion formation, we performed studies by using a disease mouse model that exhibits the features of blood stasis and stagnation.

**Methods:**

Four well-known TCHM formulae, frequently used for treating symptoms similar to endometriosis, were applied to a mouse model with human endometriosis features. Lesion growth, modulation of peritoneal immune responses, pain-associated behaviors, and endometriosis adhesion were analyzed after TCHM treatment.

**Results:**

Among the formulae, Danggui Buxue Tang (DBT) and Shaofu Zhuyu Tang (SZT) can promote blood circulation into the peritoneal cavity, resulting in increased large (LpMs) and small (SpMs) peritoneal macrophages with M1 phenotype polarization. Macrophage activation restrained lesion formation, chronic inflammation, and nerve fiber growth in the treated mice. Behavioral studies further confirmed pain-relief effects that enhanced spontaneous locomotor activity and reduced evoked mechanical hyperalgesia. Interestingly, DBT and SZT treatments can also downregulate the expression of key adhesion molecules, such as ICAM-1 and AKAP12, in the lesions, leading to reduced endometriosis adhesion in treated mice.

**Conclusion:**

These findings reveal the functional effects of TCHM formulae, especially DBT and SZT, on macrophage-mediated immune responses in the peritoneal cavity, which may benefit patients with endometriosis.

## 1. Introduction

Dysmenorrhea and irregular menstruation in women are common clinical issues in traditional Chinese medicine (TCM) and are frequently associated with periodic pain and abnormal menstrual bleeding. Although no specific term has been used in TCM, patients with these symptoms are usually diagnosed with endometriosis by Western medicine, a common disease in women of reproductive age that affects nearly 200 million people globally [1]. With endometrial tissues growing outside the uterine cavity, this disease is accompanied by chronic inflammation and associated complications such as infertility, scar/band formation, and hyperalgesia [1]. Persistent inflammatory milieus triggered by neutrophils or macrophages in a condition of high density of nerve fiber and vasculature can promote an advanced type of endometriosis, known as deeply infiltrating endometriosis (DIE), which invades other peritoneal organs [2]. Increased cytokines/chemokines and neuromediators in DIE also sensitize neurons to noxious stimuli, known as hyperalgesia, and pain can be magnified to form a pain memory in the cerebral cortex. Patients at this stage usually experience a poor quality of life that negatively affects their sexual activities and social relationships [3]. Although it is a benign gynecologic condition, women with endometriosis, especially DIE, have two to three times the risk of developing ovarian cancer, especially clear cell and endometrioid subtypes [4,5]. Thus, more attention on endometriosis screening and management is needed for better reproductive health care in women.

Currently, nonsteroidal anti-inflammatory drugs (NSAIDs) are commonly used as a first-line treatment to relieve the pain caused by endometriosis [3]. For patients with resistance to NSAIDs, hormonal treatments such as oral contraceptives, progestins (synthetic progesterones), or gonadotropin-releasing hormone (GnRH agonist) are used to delay disease progression and suppress the recurrence rate after surgical removal [6]. However, these treatments are symptomatic and not curative therapies, resulting in high recurrence rates in patients. Therefore, laparoscopic surgery is recommended if drug treatment is ineffective. However, this task could be challenging for doctors due to complications of endometriosis adhesions. These thick bands of scar tissue make the lesions and organs stick to each other, increasing the difficulty to preserve the intact functions of peritoneal organs. Approximately 40 %–80 % of patients will suffer from pelvic pain again two years after the operation [6,7]. Notably, adhesions can become increasingly serious after multiple operations and recurrences. Owing to these limitations, alternative medicine with a successful clinical history, such as TCM, may open a new direction for clinical practice and basic research.

From the viewpoint of TCM, the symptoms of endometriosis, especially period pain, are caused by obstruction or shortage in the blood or lymphatic systems of a local microenvironment (known as “qi-circulations” in TCM) [8]. As a result, traditional Chinese herbal medicine (TCHM) for treating these symptoms is usually designed based on their ability to reduce stasis and blood coagulation to promote blood circulation and maintain fluid balance in the body. Interestingly, clinical studies have revealed that TCHM-treated patients have lower surgery rates than those treated with conventional therapy [9]. Similar findings were also reported that TCHM treatment can prolong the median time to first recurrence after the surgery [10]. In particular, the levels of several inflammatory cytokines and tumor markers in the blood are reduced after TCHM treatment [11]. Recently, a meta-analysis using data from different clinical trial studies indicated a significant reduction in pelvic pain including dysmenorrhea and dyspareunia with a lower rate of hot flush and irregular vaginal bleeding three months after TCHM treatment [12]. These interesting data provide clinical evidence supporting the potential benefits of TCHM in treating patients with endometriosis. This may explain why Asian women with pelvic pain during the cycles usually seek TCHM treatment even before they are diagnosed with endometriosis.

Previous studies have shown that natural products from so called “qi-tonifying” TCHM can inhibit pro-inflammatory pathways and control the development of chronic inflammation-related diseases without any obvious side effects. A well-known mechanism of TCHM-mediated immune modulation involves inhibition of NF-κB activity and suppression of the associated downstream effectors, including a variety of pro-inflammatory cytokines/mediators and cell adhesion proteins [13,14]. Other studies also found that certain TCHMs, known for promoting blood circulation and removing stasis, can inhibit VEGF/VEGFR2 signaling cascades, leading to inhibition of lymphangiogenesis in inflammatory tissues. Since endometriosis is defined as a disease state of chronic inflammation with well-organized structures of vasculatures and lymphatic vessels [1,15], it is highly possible that treatments with “qi-promoting” TCHMs can be effective in modulating immune responses in the peritoneal cavity of patients. To test our hypothesis, a mouse endometriosis model with human-like features was used to verify the therapeutic efficacy of four well-known TCHMs designed for women with symptoms of endometriosis [16,17]. Their effects in the traditional formulae on lesion growth, inflammation regulation, pain relief, and adhesion formation were examined and discussed in this study.

## 2. Methods

### 2.1. Mouse endometriosis model with human-like features

**Fig. 1:**
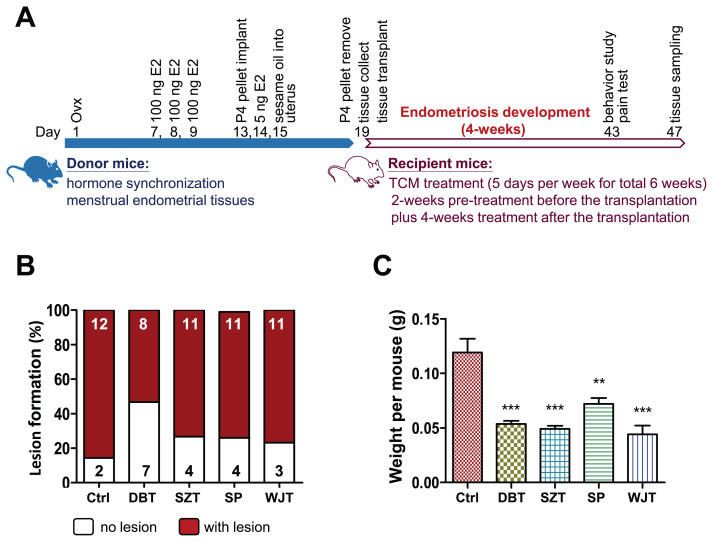
TCHM treatments in the mouse model of endometriosis in C57BL/6 mice. (A) The schematic diagram indicates the timeline of procedures in this study, including hormone synchronization and collection of decidualized tissues in the donor mice, as well as TCHM treatments and tissue transplantation in the recipient mice. The recipient mice were treated with different TCHM formulae: DBT (n = 15), SZT (n = 15), SP (n = 15), and WJT (n = 14) at the indicated drug dosages for total six weeks (Table A.1) (https://www.biomedicinej.com/cgi/editor.cgi?article=1706&window=additional_files&context=biomedicine). Corn starch-treated mice were utilized as the untreated controls (Ctrl, n = 14). (B) The lesion formation frequencies and (C) average lesion weight in TCHM-treated mice were counted and compared with the ones in control mice. Statistical differences between TCHM-treated mice and untreated controls were compared by chi-squire test in (B) or t-test in (C). The p-values were presented as *: p-value <0.05, **: p-value <0.01, and ***: p-value <0.001.

A mouse model with human endometriosis features was established according to previously described procedures [16,17]. Six-week-old female *C57BL/6J* mice were purchased from the National Laboratory Animal Center (NLAC; Taiwan). As shown in Fig. 1A, the hormone cycle was synchronized in ovariectomized donor mice on day 1 by subcutaneous injection of E2 (100 ng/mouse/day) (estradiol-17β from Sigma–Aldrich, Dorset, UK) on days 7–9, followed by progesterone treatment (P4, Sigma–Aldrich) on day 13 via a SILASTIC implant and E2 injection (5 ng/mouse) on day 14. To trigger decidualization, sesame oil was introduced into the uterus (20 μl of oil per uterine horn) on day 15, and menstrual endometrial tissues were collected from the decidualized horns on day 19, 4 h after P4 pellet withdrawal. The tissue mass (from one decidualized horn) was suspended in 0.2 mL of sterilized PBS and injected into the peritoneal cavity of the recipient mouse. Endometriotic lesions were detected in recipient mice four weeks after transplantation. Adhesions were scored based on the proportion of areas with fibrotic tissues in the intestines of diseased mice with different treatments. All protocols were performed in accordance with regulations and guidelines of the Institutional Animal Care and Use Committee of China Medical University (CMUIA-CUC Number: 2021-256 and 2021-256-1).

### 2.2. Traditional Chinese herbal medicine (TCHM) treatments in diseased mice

Two weeks before tissue transplantation, recipient mice were treated with TCHMs (five times a week for a total of six weeks) by oral gavage. The dosage of each TCHM for treating mice was estimated according to human dosage suggested by a GMP-grade TCHM provider (Sun Ten Pharmaceutical Co. Ltd., Taipei, Taiwan) (Table A.1) (https://www.biomedicinej.com/cgi/editor.cgi?article=1706&window=additional_files&context=biomedicine), under the guidance of the Food and Drug Administration (FDA)/Center for Drug Evaluation and Research (CDER) in the USA [18]. The correction factor (Km) of the dosage from humans to mouse was 12.3. Mice treated with equal amounts of corn starch were used as controls. Those with the same surgical operations, but without transplants and TCHM treatments, were used as sham healthy controls.

### 2.3. Immune cell profiling of peritoneal lavages by flow cytometry

After sacrifice on day 47, peritoneal lavages were collected from the treated mice by washing the peritoneal cavity with 5 mL serum free DMEM. Red blood cells (RBCs) in the samples were removed using RBC lysis buffer (55 mM NH_4_Cl, 12 mM NaHCO_3_ and 0.1mMEDTA), and the remaining cells were pulled down by centrifugation. Immune cells were blocked with FcR blocking reagent (rat anti-mouse CD16/CD32 Ab, clone 2.4G2, BD Biosciences, Franklin Lakes, NJ) for 2 h, followed by overnight staining on ice with a combination of antibodies (Table A.2) (https://www.biomedicinej.com/cgi/editor.cgi?article=1706&window=additional_files&context=biomedicine). On the second day, the staining signals were analyzed using an LSR II Flow Cytometer (BD Biosciences) and immune cell populations were defined based on the gating strategy shown in Fig. A.1 (https://www.biomedicinej.com/cgi/editor.cgi?article=1706&window=additional_files&context=biomedicine). The collected data were further analyzed using FlowJo v.10 software (FlowJo LLC., Ashland, OR).

### 2.4. Tissue section staining on endometriotic lesions

Endometriotic lesions were collected on day 47 and sent for tissue-block preparation. Immunohistochemistry (IHC) and immunofluorescence (IF) staining were performed on tissue sections according to procedures described in previous studies [16,17,19]. Detailed information regarding the antibodies and their dilutions can be found in Table A.3 (https://www.biomedicinej.com/cgi/editor.cgi?article=1706&window=additional_files&context=biomedicine). IHC and IF images were taken by using an IX83 fluorescent microscope (Olympus Corp., Shinjuku, Japan), and the area of positive staining was quantified using Image-Pro Premier (Media Cybernetics Inc., Rockville, MD).

### 2.5. Behavioral assessments

Spontaneous (grooming and activity) and evoked (mechanical hyperalgesia measured using von Frey filaments) behaviors of the treated mice were monitored four days before sacrifice in a blinded fashion. During the assessment, all animals were acclimatized to the apparatus and handled prior to the beginning of behavioral analysis. Detailed information about the behavioral assessments were described in a previous study [17].

### 2.6. Immunoassay for protein quantification

Peritoneal fluids were collected for immune cell profiling. Protein concentrations of 16 common inflammation-related cytokines in the samples were quantified using a multiplex immunoassay supported by the Inflammation Core Facility at Academia Sinica, Taiwan. To detect the levels of β-NGF and IGF-1 in the fluids, sandwich enzyme-linked immunosorbent assay (ELISA) kits were purchased from Thermo Fisher Scientific (mouse β-NGF: EM9RB; mouse IGF-1: EMIGF1). ELISA was performed according to the manufacturer’s protocol provided by the company. Colorimetric signals were measured at 650 nm with an iMark microplate reader (Bio-Rad Lab., Hercules, CA), and the absolute concentrations were normalized to standard curves of the recombinant proteins provided by kits.

### 2.7. Real-time quantitative-PCR (qPCR) for gene expression analysis

RNA samples were extracted from several tissues of the treated mice, including endometriotic lesions, peritoneal membranes, spinal cords, and posterior insula, using an RNA extraction kit (FairBiotech, Taoyuan, Taiwan). The RNA samples were immediately subjected to cDNA synthesis using a High-Capacity cDNA Reverse Transcription Kit (Thermo Fisher Scientific). Real-time qPCR was performed in triplicate to measure the expression of inflammatory cytokines and neurotrophic factors. The detailed primer sequences for target genes are listed in Table A.4 (https://www.biomedicinej.com/cgi/editor.cgi?article=1706&window=additional_files&context=biomedicine). Cycling conditions for qPCR reactions included an initial denaturation at 95 °C for 1 min, followed by 45 cycles of 95 °C for 15 s, 60 °C for 15 s, and 68 °C for 15 s. GAPDH expression levels were used as internal controls for data normalization.

### 2.8. Statistical analysis

Statistical analysis in this study was carried out using SPSS software (version 14.0; SPSS Inc. Chicago, IL), or GraphPad Prism (GraphPad Software, La Jolla, CA). The t-test was used for the comparison between two groups, while Wilcoxon and one-way ANOVA tests were used for the study with more than two groups. Tukey’s test was used to determine significant differences between multiple comparisons. The data are presented as mean ± S.D., and a *p*-value less than 0.05 was considered statistically significant.

## 3. Results

### 3.1. Traditional Chinese herbal medicines (TCHMs) reduce the sizes of endometriotic lesions in diseased mice

A mouse model with human endometriosis features was established in 6-week-old female *C57BL/6* mice for this study (Fig. 1A). As defined in previous studies, endometriotic lesions could be detected in recipients 4 weeks after transplantation [16,17]. Both epithelial (anti-cytokeratin) and stromal (anti-vimentin) cell compartments could be found in lesions with well-developed vasculatures (anti-CD31), lymphatic vessels (anti-LYVE-1), and nerve fibers (anti-PGP9.5) (Fig. A.2) (https://www.biomedicinej.com/cgi/editor.cgi?article=1706&window=additional_files&context=biomedicine). Two weeks before transplantation, the recipient mice were pretreated with different traditional Chinese herbal medicines (TCHMs) (n = 15 in each group) or an equal amount of corn starch as controls for a total of six weeks (Fig. 1A). The TCHMs included Dang-Gui Bu-Xue Tang (DBT), Shao-Fu Zhu-Yu Tang (SZT), Shi-Xiao Powder (SP), and Wen Jing Tang (WJT), all were known as “qi-promoting” TCHMs. Mice in the sham healthy group underwent the same surgical operations, but without transplants and herbal treatments. Compared to controls, DBT-treated mice showed a trend to reduce the incidence rate of endometriosis ( *p* = 0.0596) (Fig. 1B). On the other hand, all the four TCHMs can significantly reduce the lesion sizes as compared to corn starch treated controls (Fig. 1C). No obvious functional damage in the liver and kidneys were observed after the herbal treatments (Fig. A.3) (https://www.biomedicinej.com/cgi/editor.cgi?article=1706&window=additional_files&context=biomedicine). Thus, our data confirm the therapeutic benefits of treating diseased mice with these four TCHMs.

### 3.2. TCHMs can modify the peritoneal immune milieu

**Fig. 2:**
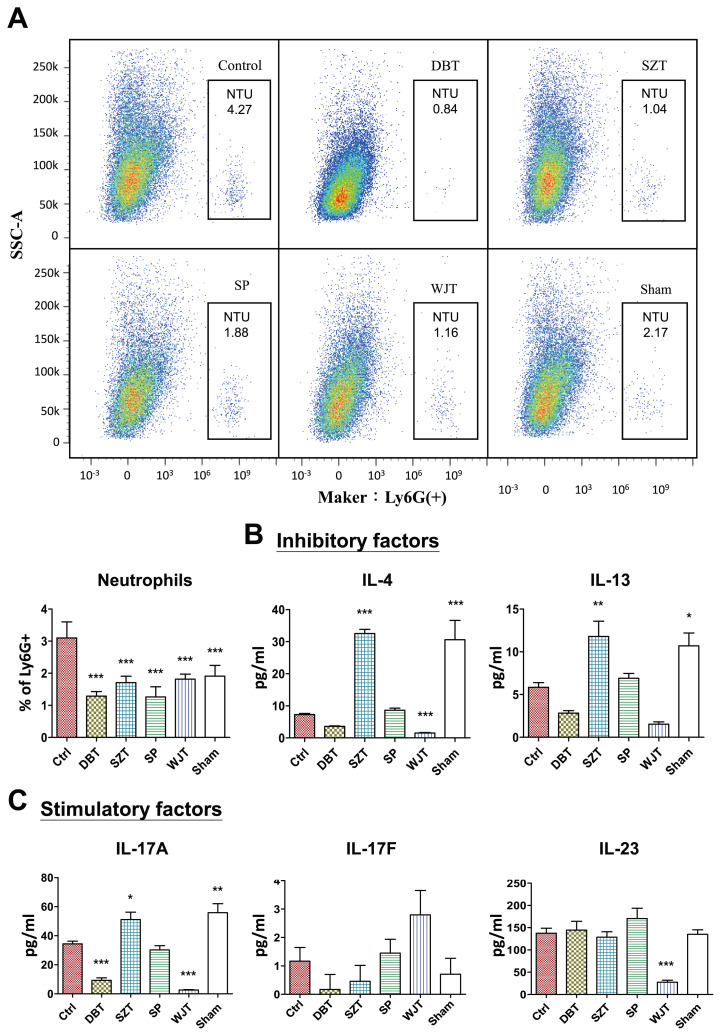
Regulation of neutrophil-mediated inflammation by TCHMs in the peritoneal cavity of mice with induced endometriosis. (A) Multicolor flow cytometry was used to assess myeloid cell populations in the peritoneal fluid of TCHM-treated mice with induced endometriosis by using the gating strategy shown in Fig. A.1 (https://www.biomedicinej.com/cgi/editor.cgi?article=1706&window=additional_files&context=biomedicine). The depicted cellular gating is representative of individual treatments. Neutrophils (NTU, Ly6G+) in the lavages were quantified and compared among different groups. The bar chart summarized the calculated amount of neutrophils in each group. The levels of (B) inhibitory factors (IL-4 and IL-13) and (C) stimulatory factors (IL-17A, IL-17F, and IL-23) in the peritoneal fluid for neutrophil recruitment or expansion were measured by multi-plex immunoassay. Statistical differences between TCHM-treated mice and untreated controls were compared by using t-test. Sham healthy mice served as the negative controls. The p-values were presented as *: p-value <0.05, **: p-value <0.01, and ***: p-value <0.001.

Patients with endometriosis have been known to experience disruptions in immune cell function [1,15]. Therefore, we investigated the possible effects of TCHMs on peritoneal immune milieu. Peritoneal fluids were collected from control and herb-treated mice on day 47 (Fig. 1A). Immune cells inside the fluids were pulled down for antibody staining (Table A.2) (https://www.biomedicinej.com/cgi/editor.cgi?article=1706&window=additional_files&context=biomedicine) and then subjected to flow cytometry analyses using the gating strategy shown in Fig. A.1 (https://www.biomedicinej.com/cgi/editor.cgi?article=1706&window=additional_files&context=biomedicine). Neutrophil-mediated chronic inflammation was enhanced (3.10 ± 0.50 % of total leukocytes) in the peritoneal cavity of controls as compared to mice in sham healthy group (1.91 ± 0.33 %) (Fig. 2A). Herbal treatment significantly reduced neutrophil levels, similar to those in sham healthy mice (Fig. 2A). Notably, the levels of IL-4 and IL-13, inhibitory factors for neutrophils, in SZT-treated mice were maintained as high as those in healthy mice, suggesting restrictions on neutrophil activation and expansion (Fig. 2B). For the stimulatory factors, IL-17A and IL-17F levels appeared to be balanced on a seesaw in the peritoneal cavity, suggesting their distinct but also collaborative roles in regulating neutrophil recruitment and inflammation [20]. For example, WJT-treated mice showed the lowest IL-17A levels but the highest IL-17F levels in their peritoneal fluids. Among the herbal formulae, DBT was the only one that reduced both IL-17A and IL-17F levels (Fig. 2C). Furthermore, WJT treatment significantly reduced IL-23 levels in the peritoneal fluids of treated mice (Fig. 2C).

### 3.3. TCHMs influence macrophage dynamics in the peritoneal cavity

**Fig. 3:**
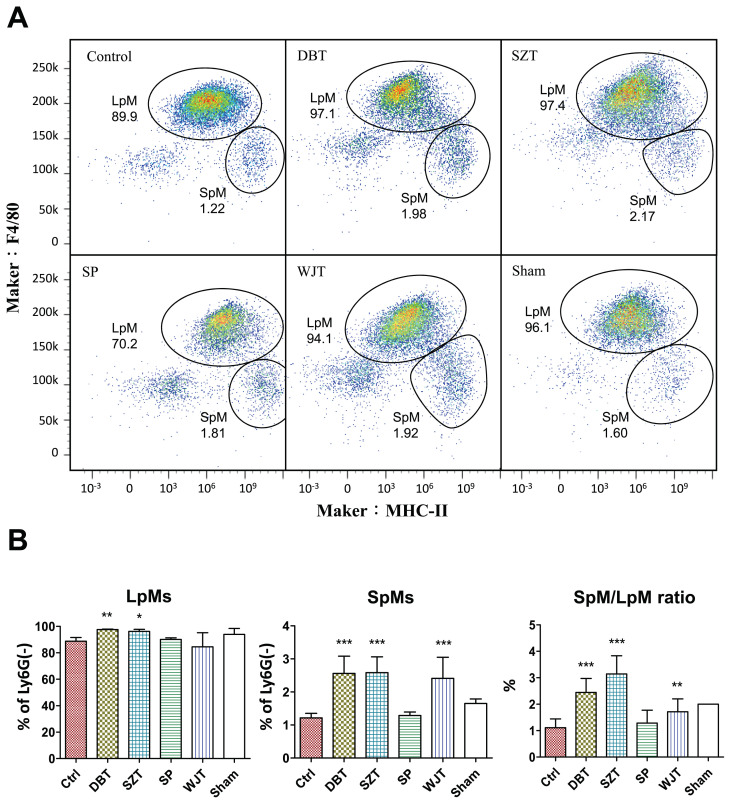
Regulation of macrophage populations by TCHMs in the peritoneal cavity of mice with induced endometriosis. (A) Multicolor flow cytometry was applied to analyze macrophage subsets by using the gating strategy shown in Fig. A.1 (https://www.biomedicinej.com/cgi/editor.cgi?article=1706&window=additional_files&context=biomedicine). Monocytes (CD45.2^+^/Ly6G^−^) expressing higher F4/80 levels were considered as large peritoneal macrophages (LpM) and those expressing higher MHC-II whiles lower F4/80 were considered as small peritoneal macrophages (SpM). The depicted cellular gating is representative of individual treatments. (B) The bar charts summarized the calculated amounts of LpM (left) or SpM (middle) and the ratios (SpM/LpM) of these two (right) in each group. Statistical differences between TCHM-treated mice and untreated controls were compared by using t-test. Sham healthy mice served as the negative controls. The p-values were presented as *: p-value <0.05, **: p-value <0.01, and ***: p-value <0.001.

In addition to neutrophils, small (SpM) and large (LpM) peritoneal macrophages have also been defined as key players in modulating immune microenvironment in peritoneal cavity [21,22]. By applying the gating strategy shown in Fig. A.1 (https://www.biomedicinej.com/cgi/editor.cgi?article=1706&window=additional_files&context=biomedicine), the proportions of these two macrophage subsets in peritoneal fluids were analyzed (Fig. 3A). LpM levels were found to be slightly increased in mice treated with DBT and SZT, whereas SpM levels were strongly enhanced by treatments with DBT, SZT, and WJT. Significantly, the proportional ratios between SpM and LpM (SpM/LpM) were enhanced in DBT-and SZT-treated mice, and were even higher than the ratios in healthy mice (Fig. 3B). Since most SpMs are generated from hematopoietic stem cells in bone marrow [21,22], our data may support the viewpoint that more circulating monocytes were recruited into the peritoneal cavity from peripheral vascular or lymphatic systems by those “qi-promoting” herbal formulae.

To confirm this, we focused on cells isolated from peritoneal lavage that showed the feature of CD45^+^/Cd11b^+^/Ly6G^−^ (monocyte-macrophage lineage) and subdivided them into four populations (Q1 to Q4) based on their expression levels of F4/80 and Ly6C (Fig. A.4A) (https://www.biomedicinej.com/cgi/editor.cgi?article=1706&window=additional_files&context=biomedicine) [23]. Consistent with the findings shown in Fig. 3, DBT and SZT were unique formulae that elevated LpMs (Q1) and SpMs (Q3) in the peritoneal cavity of treated mice (Fig. A.4B) (https://www.biomedicinej.com/cgi/editor.cgi?article=1706&window=additional_files&context=biomedicine). In addition, monocyte-derived macrophages (MDMs) (Q2) were also significantly enhanced by DBT and SZT, which was not observed in mice treated with other herbal formulae or mice in the sham healthy group (Fig. A.4B) (https://www.biomedicinej.com/cgi/editor.cgi?article=1706&window=additional_files&context=biomedicine). Compared to the controls without TCHM treatment, mice treated with herbal formulae or mice in the sham healthy group showed higher monocytes (Q4) in the peritoneal cavity. According to the dynamic profiles, we may conclude that healthy mice or diseased mice with effective TCHM treatments such as DBT and SZT show better micro-circulations (Q2+Q3+Q4) from peripheral vascular or lymphatic systems into the peritoneal cavity.

### 3.4. TCHMs enhance M1-macrophage polarization and attenuate markers of neurotropic inflammation

**Fig. 4:**
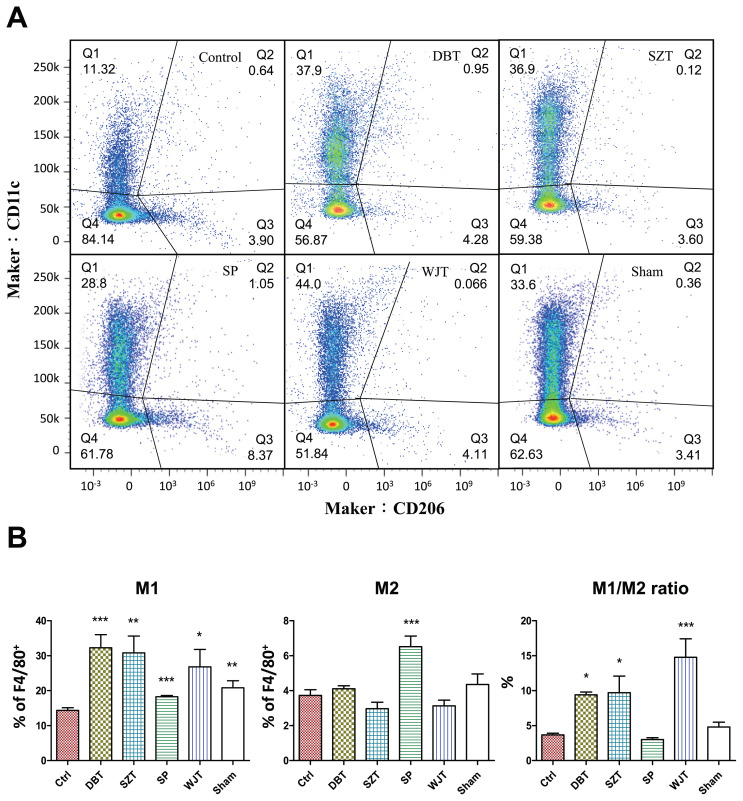
Regulation of macrophage polarization by TCHMs in the peritoneal cavity of mice with induced endometriosis. (A) Multicolor flow cytometry was applied to analyze macrophage polarization in the peritoneal cavity of TCHM-treated mice with induced endometriosis by using the gating strategy shown in Fig. A.1 (https://www.biomedicinej.com/cgi/editor.cgi?article=1706&window=additional_files&context=biomedicine). Macrophage subtypes (F4/80^+^ monocytes) were further analyzed by detecting the expression of CD11c for type-1 macrophages (M1) or CD206 for type-2 macrophages (M2). The depicted cellular gating is representative of individual treatments. (B) The bar charts summarized the calculated amounts of M1 (left) or M2 (middle) and the ratios of these two (M1/M2, right) in each group. Statistical differences between TCHM-treated mice and untreated controls were compared by using t-test. Sham healthy mice served as the negative controls. The p-values were presented as *: p-value <0.05, **: p-value <0.01, and ***: p-value <0.001.

To determine the possible roles of TCHMs in modulating macrophage polarization, M1 (F4/80^+^, CD11c^+^) and M2 (F4/80^+^, CD206^+^) subtypes were analyzed using the gating strategy shown in Fig. A.1 (https://www.biomedicinej.com/cgi/editor.cgi?article=1706&window=additional_files&context=biomedicine). As shown in Fig. 4A, reduction of the M1 population was a key feature for corn starch-treated diseased mice as compared to the sham healthy mice, indicating chronic inflammatory conditions in the peritoneal cavity. After TCHM treatment, increased M1 polarization was observed, especially in mice treated with DBT, SZT, and WJT, resulting in an increased M1/M2 ratio (Fig. 4A and B). Our data from Fig. A.4 (https://www.biomedicinej.com/cgi/editor.cgi?article=1706&window=additional_files&context=biomedicine) and Fig. 4 suggest that DBT and SZT treatments can recruit more circulating monocytes into the peritoneal cavity and promote macrophage differentiation into M1-inflammatory macrophages, which can subsequently target and remove endometriotic lesions more efficiently.

**Fig. 5:**
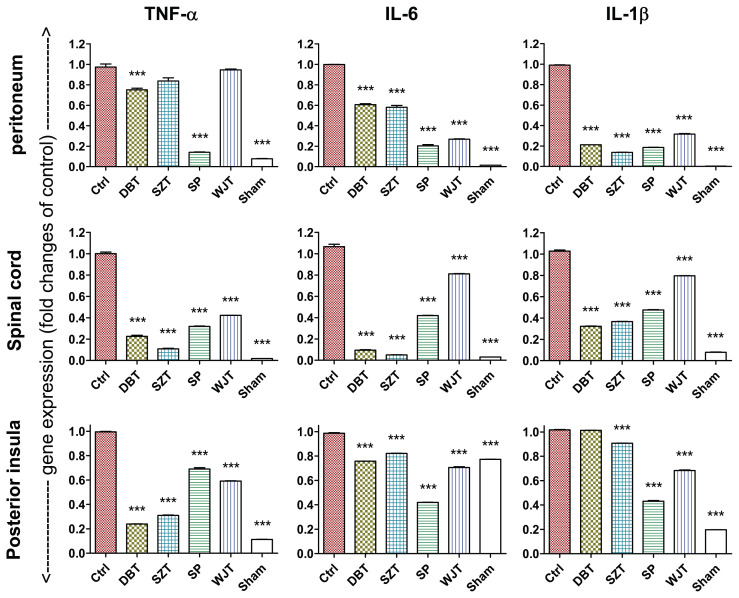
Regulation of inflammatory mediators in peritoneal tissue, spinal cord and posterior insula by TCHM treatments in mice with induced endometriosis. RNA samples were extracted from peritoneal tissues, spinal cords and posterior insula in the brains of experimental mice and subjected to cDNA synthesis. Real-time qPCR was performed on those cDNA samples (n = 6 for each group) to detect expression levels of TNF-α, IL-6, and IL-1β in different tissues. The data were normalized with average levels of the controls. Statistical differences between TCHM-treated mice and untreated controls were compared by using t-test. Sham healthy mice served as the negative controls. The p-values were presented as *: p-value <0.05, **: p-value <0.01, and ***: p-value <0.001.

To determine the suppressive effects of TCHMs on chronic inflammation and neuropathic pain, we performed qPCR to estimate the expression levels of three key inflammatory cytokines in the peritoneum, spinal cord, and posterior insula of treated mice [3,24]. TNF-α plays a pivotal role at both peripheral and central levels of sensitization [23,25]. We found that DBT and SZT significantly reduced TNF-α levels in the spinal cord and posterior insula of treated mice (Fig. 5), whereas the levels were still high in the peritoneum, probably because of more active M1-macrophages in the treated mice (Fig. 4A and B) [26,27]. As being a cytokine involved in chronic inflammation, IL-6 has been defined as an emerging regulator of pathological pain [28]. We found that all TCHMs downregulated IL-6 expression in the peritoneum, spinal cord, and posterior insula of treated mice (Fig. 5). Since IL-6 is a key regulator for M2 macrophage polarization [29], these data may provide additional evidence from a different aspect that DBT and SZT treatments guide the polarization of LpMs and SpMs to show more phenotypes as M1-inflammatory macrophages. Furthermore, all TCHMs downregulated IL-1β expression in the peritoneum and spinal cord (Fig. 5). However, its levels in the posterior insula were difficult to reduce with these formulae, which may reflect its potent role in controlling neuronal activity in central nervous system, leading to memory formation of hyperalgesia [3,30]. On the other hand, WJT and SP may be more effective for pain management because they can strongly suppress IL-1β expression in the posterior insula of the treated mice (Fig. 5).

### 3.5. TCHMs suppress hyperalgesia in mice with endometriosis

**Fig. 6:**
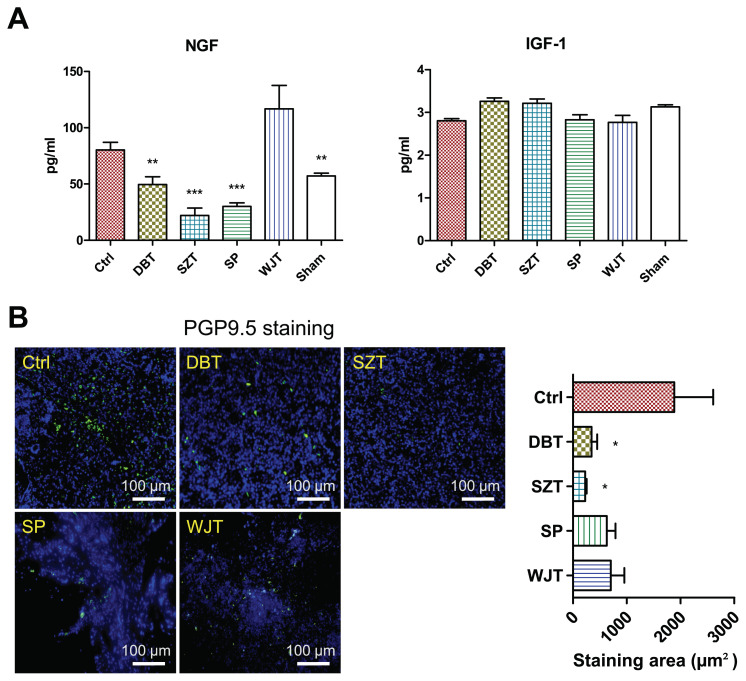
Regulation of nerve fiber growth in endometriotic lesions by TCHMs in mice with induced endometriosis. (A) Protein levels of NGF (left) and IGF-1 (right) in the peritoneal fluid of experimental mice were analyzed by sandwich ELISA. Protein concentrations were normalized with standard curves of the recombinant proteins. (B) IF staining was performed to detect the presence of nerve fibers (PGP9.5^+^) in the endometriotic lesions from mice with different treatments (left). The fluorescence area was averaged by using data from 10 independent tissue sections (right). Statistical differences between TCHM-treated mice and untreated controls were compared by using t-test. Sham healthy mice served as the negative controls. The p-values were presented as *: p-value <0.05, **: p-value <0.01, and ***: p-value <0.001.

Endometriosis-associated hyperalgesia features nerve growth factor expression and nerve infiltration, making a connection between endometriotic lesions and the surrounding nerves [3,31]. To better understand therapeutic effects of these herbal formulae on nerve fiber growth, ELISA was performed to analyze protein levels of NGF and IGF-1 in the peritoneal fluids, two neurotropic and sensitizing factors secreted from endometriosis-associated macrophages with M2 phenotypes [23,32]. Consistent with the modulatory activity on macrophage differentiation and M1-polarization, treatments with DBT, SZT, or SP reduced NGF levels in the peritoneal cavity of treated mice compared to untreated controls (Fig. 6A). IF staining for PGP9.5, a neuron-specific marker, further indicated reduced nerve fiber density within the endometriotic lesions compared to lesions from untreated controls (Fig. 6B). In this study, we found limited suppressive effects on IGF-1 secretion after TCHM treatments.

**Fig. 7:**
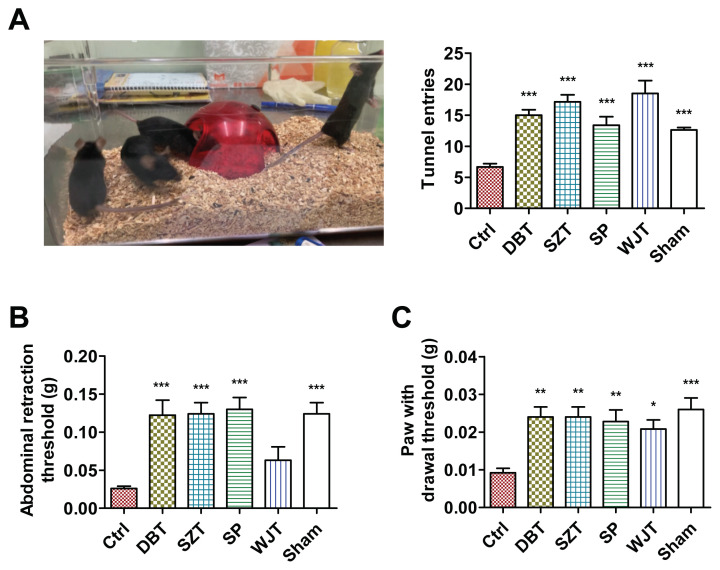
Impacts of TCHM treatments on pain relief in mice with induced endometriosis. Behavioral study was performed to monitor (A) tunnelentering activity within 5 min in mice treated with different formulae (left) and presented as a bar chart (right). Touch test (von Frey) was also performed on (B) abdomen and (C) hind paws of mice to quantify pain-sensitivity. The corn starch-treated mice were utilized as the untreated controls whereas sham mice were utilized as healthy controls. The data were averaged from three independent observations (n = 15 for each group). Statistical differences between TCHM-treated mice and untreated controls were compared by using one-way ANOVA (Kruskal–Wallis test). The p-values were presented as *: p-value <0.05, **: p-value <0.01, and ***: p-value <0.001.

To confirm the potential impacts on sensory behaviors of treated mice, we monitored spontaneous activities by counting the number of entries through an enrichment tunnel within 5 min (Fig. 7A, *left* panel). Our data indicated an overall improvement in locomotor activities by treating diseased mice with TCHMs (Fig. 7A, *right*). The tunnel-entering activities of the treated mice were similar to those of sham healthy mice. To confirm these results, evoked mechanical hyperalgesia on the abdomen and hind paws was measured using von Frey filaments. Except for WJT treatment, in which the treated mice showed higher nerve fiber density (Fig. 6B), other herbal formulae significantly reduced pain sensitivity in the abdomen of the treated mice (Fig. 7B). Regarding the pain test on hind paws, all herbal formulae were found to be effective in reducing pain sensitivity (Fig. 7C).

### 3.6. TCHMs reduce adhesion potentials in mice with endometriosis

**Fig. 8:**
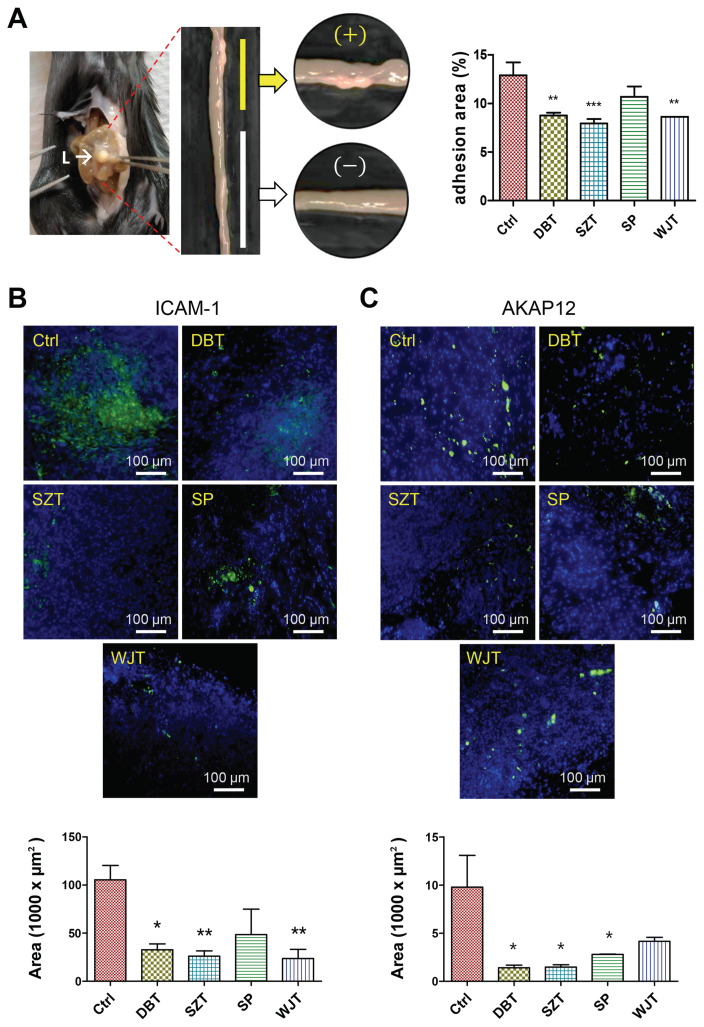
Regulation of endometriosis adhesions by TCHMs in mice with induced endometriosis. (A) Mice with induced endometriosis show adhesions on the intestine and the “L” indicates the endometriotic lesion (left). The bar chart summarized the ratios between fibrotic and smooth regions along with the intestines of experimental mice treated with different TCHMs (right). IF staining was performed to detect the expression levels of (B) ICAM-1 and (C) AKAP12, two important adhesion molecules, in the endometriotic lesions from mice with different treatments. The fluorescence area was averaged by using data from 10 independent tissue sections. Statistical differences between TCHM-treated mice and untreated controls were compared by using t-test. Sham healthy mice served as the negative controls. The p-values were presented as *: p-value <0.05, **: p-value <0.01, and ***: p-value <0.001.

Endometriosis-associated adhesions cause challenges in clinical management because they can lead to complications in patients [33]. In our mouse study, we frequently found intestinal clamping in diseased mice, which showed a different phenotype from those in healthy mice (Fig. 8A, *left*). Therefore, we were interested in determining the potential benefits of TCHM treatments on endometriosis adhesions. First, we measured the areas of fibrous tissues on the intestines (Fig. 8A, *left*), and our data indicated significant reductions in adhesion formation in the intestine of diseased mice treated with DBT, SZT, and WJT (Fig. 8A, *right*). With this evidence, we analyzed the expression levels of two well-known adhesion molecules, ICAM-1 and AKAP12, by IF staining. These two molecules have been previously suggested as potential biomarkers for invasive endometriosis adhesions or post-surgical adhesions [33,34]. As shown in Fig. 8B and C, significant reductions in these two adhesion molecules were found in the lesions of mice treated with DBT and SZT. The expression of ICAM-1 and AKAP12 was also responsive to WJT and SP treatments, suggesting the therapeutic benefits of TCHMs in reducing endometriosis adhesions.

## 4. Discussion

In this study, we provide molecular evidence confirming the therapeutic effectiveness of four well-known TCHMs in treating endometriosis using a disease mouse model with human-like features. Among them, DBT and SZT were further identified as the most active formulae that not only reduced lesion sizes and neutrophil-mediated inflammation (Figs. 1 and 2) but also showed modulatory effects on macrophage profiles, differentiation, polarization, and the mediated inflammation by promoting vascular and lymphatic circulation (Figs. 3–5, Fig. A.4 (https://www.biomedicinej.com/cgi/editor.cgi?article=1706&window=additional_files&context=biomedicine)). Consequently, endometriosis-associated pain (Figs. 5–7) and adhesions (Fig. 8) were also reduced by DBT and SZT treatments. Given the absence of effective drugs for treating endometriosis, our data suggest the potential value of TCHMs, especially DBT and SZT, for clinical practice. SP and WJT treatments also reduced lesion size (Fig. 1) and local inflammation (Figs. 2 and 4) with enhanced locomotor activity and pain tolerance (Fig. 7) in diseased mice. However, they played little role in macrophage modulation; thus, nerve fiber intensity was still relatively higher in treated mice (Fig. 6). Interestingly, IL-1β levels in the posterior insula of diseased mice, the key player in neuropathic pain in central nervous system [30], were significantly downregulated by SP and WJT treatments, suggesting their roles in reducing central sensitization [24], which deserves more detailed investigation.

Traditionally, the theoretical basis of TCHM treatment is syndrome differentiation by suggesting appropriate prescriptions for patients with different symptoms. Since irregular bleeding between cycles is an important symptom for patients with endometriosis, DBT is considered an effective formula for treating endometriosis due to its well-known ability to produce more red blood cells [35,36]. Phthalide derivatives from *Angelica sinensis*, one major herb in DBT and SZT, have also been shown to promote oxygen transportation via allosteric stabilization of hemoglobin with oxygen [37]. In addition, this herb is known to be a fair source of vitamin B12, folate, and iron, which help our bodies generate more hemoglobin from foods [38]. These findings suggest the therapeutic benefits of DBT treatment by reducing cellular hypoxia in deeper tissues such as endometriotic lesions. Accumulating evidence has shown that hypoxia plays critical and diverse roles in endometriosis progression, such as increased adhesion ability, dysregulation of estrogen biosynthesis, aberrant production of pro-inflammatory cytokines, enhanced angiogenesis, and suppression of immune functions [39,40]. Further investigation is helpful to confirm whether the amount and activity of hypoxia-inducible factors, such as COUP-TFII [41], could be directly influenced by DBT in the endometriotic lesions of treated mice.

On the other hand, SZT consists of ten crude herbs, which show strong anti-coagulant, antihyperalgesia, and anti-inflammatory activities in the human body. This formula is used to treat patients with blood stasis syndrome, especially those with gynecological diseases, such as endometriosis [42]. With several major bioactive components identified, SZT is believed to participate in the regulation of diverse physiological functions, including thrombosis, blood flow (micro-)circulation, systemic inflammation, uterine hypercontraction/muscle constriction, and vasodilation [42,43]. Notably, meta-analyses based on different clinical studies have suggested that SZT is the most effective TCHM for pain management in women with endometriosis or dysmenorrhea [44]. The major mechanism associated with pain relief was the reduction in the expression of inflammatory genes, including IL-1β, TNF-α, IL-10, IL-2, IL-6, IL-12, CCL2, and Serpine1 [45], some of which could be also confirmed in this study (Fig. 5). Furthermore, a metabolomics study confirmed alterations in the corrections of altered metabolism of fatty acids and amino acids in high-fat diet-induced obese mice by SZT treatment. Since metabolic dysfunction is an emerging hallmark during endometriosis progression and has been suggested as a potential therapeutic target [46], correction of altered metabolism by SZT may provide another direction for future studies on endometriosis treatment.

In addition to the regulation of inflammatory cytokine profiles, our study revealed potent modulatory activity of DBT and SZT on macrophage recruitment, differentiation, and M1/M2 polarization. Macrophages are extremely sensitive to external stimuli, thus boosting blood and lymphatic circulation by TCHMs could be an important driving force to reconstitute the microenvironment in the peritoneal cavity of diseased mice. Consistent with syndrome differentiation in TCM [8], diseased mice showed fewer circulating monocytes than healthy mice (Fig. A.4B) (https://www.biomedicinej.com/cgi/editor.cgi?article=1706&window=additional_files&context=biomedicine), indicating obstruction in blood or lymphatic circulatory systems of the local region. Interestingly, all these four “qi-promoting” TCHMs can significantly increase the numbers of circulating immune cells or monocytes to the levels as shown in healthy mice. Among them, DBT and SZT were found to be the most efficient formulae for guiding the activation and polarization of macrophages (Fig. 4 and Fig. A.4 (https://www.biomedicinej.com/cgi/editor.cgi?article=1706&window=additional_files&context=biomedicine)).

As discussed above, certain components of DBT and SZT may participate directly or indirectly in these cellular processes. For example, an acidic polysaccharide fraction isolated from the roots of *Angelica sinensis* has been defined as a strong stimulator to activate macrophages via TLR4-mediated signaling and trigger M1 polarization with enhanced phagocytosis activity via upregulation of iNOS and TNF-α production [47,48]. Astragaloside IV (*Astragalus membranaceus* in DBT) was also reported to significantly reduce inflammation and lesion activity in a murine endometriosis model by suppressing the TLR4/NF-κB signaling pathway and decreasing pro-inflammatory cytokines [49]. Several key flavonoids such as quercetin, isorhamnetin, kaempferol, D-catechin, naringenin, and amentoflavone from DBT and SP are implicated in modulating hypoxia, vascular function, and inflammation via pathways involving VEGFA, AKT1, EGFR, PTGS2 (COX-2), and MMP9 [50,51]. Furthermore, quercetin, kaempferol, wogonin, β-sitosterol, and stigmasterol are five top bioactive compounds in WJT [52], which are thought to impact both inflammation and the endocrine system through modulating IL6 and ESR1 signaling. More efforts are needed to individually define these active substances for more detailed mechanistic studies [53].

Several studies have shown that endometrium-resident macrophages (LpMs) predominantly present the M2 phenotype, and the abnormal endometriotic milieu usually establishes a microenvironment for M2 polarization, promoting lesion growth, nerve fiber infiltration, and stromal cell adhesion and invasion [32,54]. Therefore, macrophage reprogramming and replacement are considered therapeutic strategies against endometriosis [22,32]. Through the recruitment of more circulating monocytes, DBT and SZT treatments not only increased SpM levels, but also elevated LpMs (Fig. 3), suggesting the replacement of LpMs from circulating monocytes in the peritoneal cavity. Consistent with our findings, macrophages derived from circulating monocytes express more Sema3A and its receptors, such as NRP-1, which transmit signals favoring M2-to-M1 reprogramming, especially under conditions of reduced hypoxia [32,55]. These findings may provide the basis to explain why DBT and SZT treatments can show therapeutic benefits for pain relief and adhesion removal in diseased mice (Figs. 5–8).

## 5. Conclusion

In summary, our study provides clear molecular evidence that certain “qi-promoting” TCHM formulae, such as DBT and SZT, show therapeutic benefits in endometriosis treatment by modulating the immune microenvironment of peritoneal cavity. In particular, macrophage differentiation from circulating monocytes and M1 polarization can be significantly enhanced by these treatments, which are associated with smaller lesion sizes, reduced chronic inflammation, less nerve fiber growth, pain relief, and fewer adhesions. New TCHM formulae can be accordingly defined for endometriosis treatment by combining herbal medicines that show “qi-promoting” activity and contain active components triggering M1 polarization.

## Supplementary Information







## Data Availability

Data from the experiments presented in this study are included in this published article and its Supporting Information files. Other data generated or analyzed during this study are available from the corresponding authors upon request.
