# Supplementary material for: Traditional Chinese herbal medicines suppress endometriosis development through modulating macrophage-mediated immune responses in the peritoneal cavity
Source: Biomedicine (Taipei). 2026 Jun 1;16(2):35–51. doi: 10.37796/2211-8039.1706 (PMC13387404; doi:10.37796/2211-8039.1706)
Supplement: Supplementary file 1 [file bmed-16-02-035-s001.pdf]

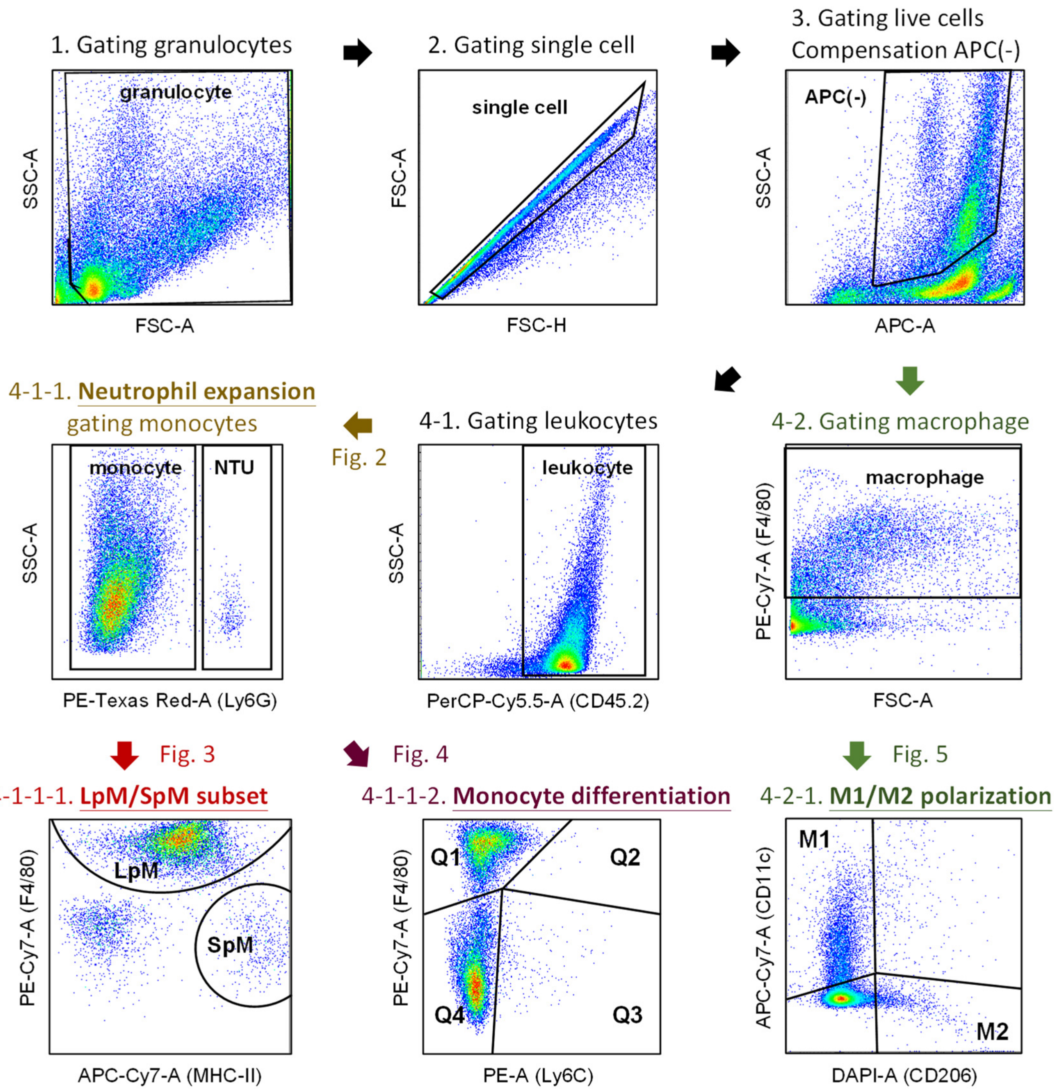

Figure S1. The gating strategy utilized for flow cytometry study. Myeloid cell populations in peritoneal lavages from treated mice were analyzed by multicolor flow cytometry. After staining with a combination mixture of antibodies shown in Table S2, live single granulocytes (APC^{-}) were filtered out through gating step 1 to step 3. Leukocytes were selected by detecting CD45.2 expression (step 4-1), and neutrophil expansion/recruitment in total leukocytes (shown in Fig. 2) were quantified by detecting Ly6G expression (step 4-1-1). Macrophage transition from small (SpM) to large (LpM) peritoneal macrophages (in Fig. 3) were further analyzed by detecting monocytes ($Ly6G^{-}$ cells in step 4-1-1) expressing F4/80 (for LpM) or MHC-II (for SpM) (step 4-1-1-1). Monocyte circulation and differentiation (in Fig. 4) were analyzed by detecting the expression of F4/80 and Ly6C (step 4-1-1-2) in total monocytes ($Ly6G^{-}$ cells in step 4-1-1). Furthermore, macrophage polarization between type-1 (M1) and type-2 (M2) (shown in Fig. 5) were analyzed by detecting macrophages ($F4/80^{+}$ cells in step 4-2) expressing CD11c (for M1) or CD206 (for M2) (step 4-2-1).

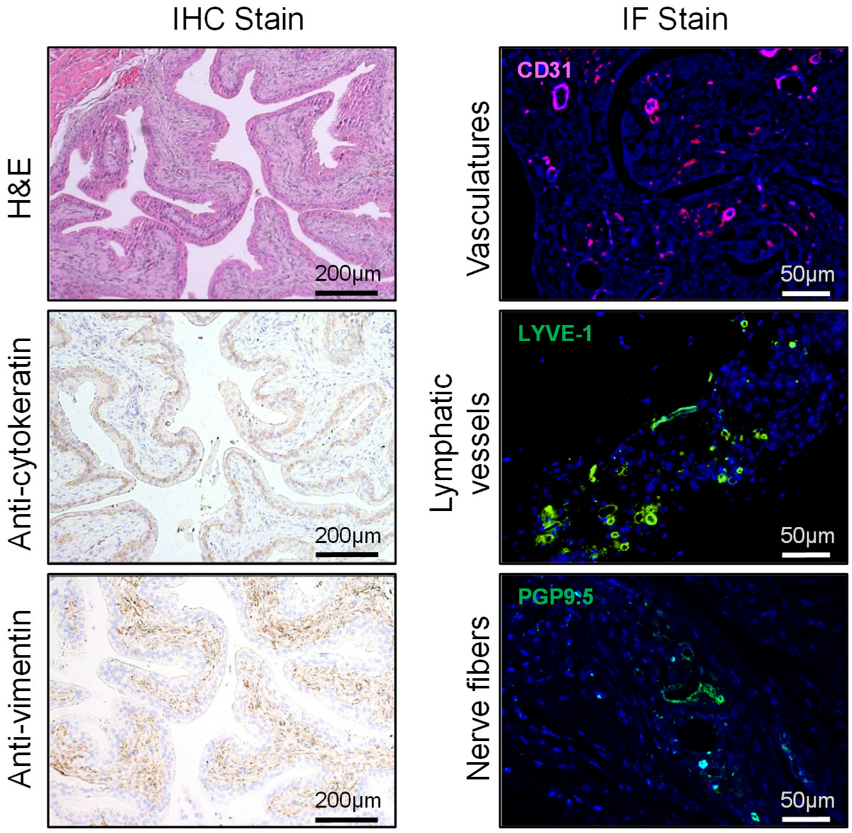

Figure S2. Establishment of endometriotic lesions in *C57BL/6* mice. Four weeks after the transplantation of decidualized tissues, lesion-like tissues were collected from the peritoneal cavity of the mice. Tissue sections were prepared to detect the presence of stroma (anti-vimentin) or glands (anti-cytokeratin) in the collected lesions by IHC staining. The parallel H&E image was utilized as the reference. To study the human features, IF staining was performed to confirm the well-developed vasculatures (anti-CD31), lymphatic vessels (anti-LYVE-1) and nerve fiber (anti-PGP9.5) in the defined lesion.

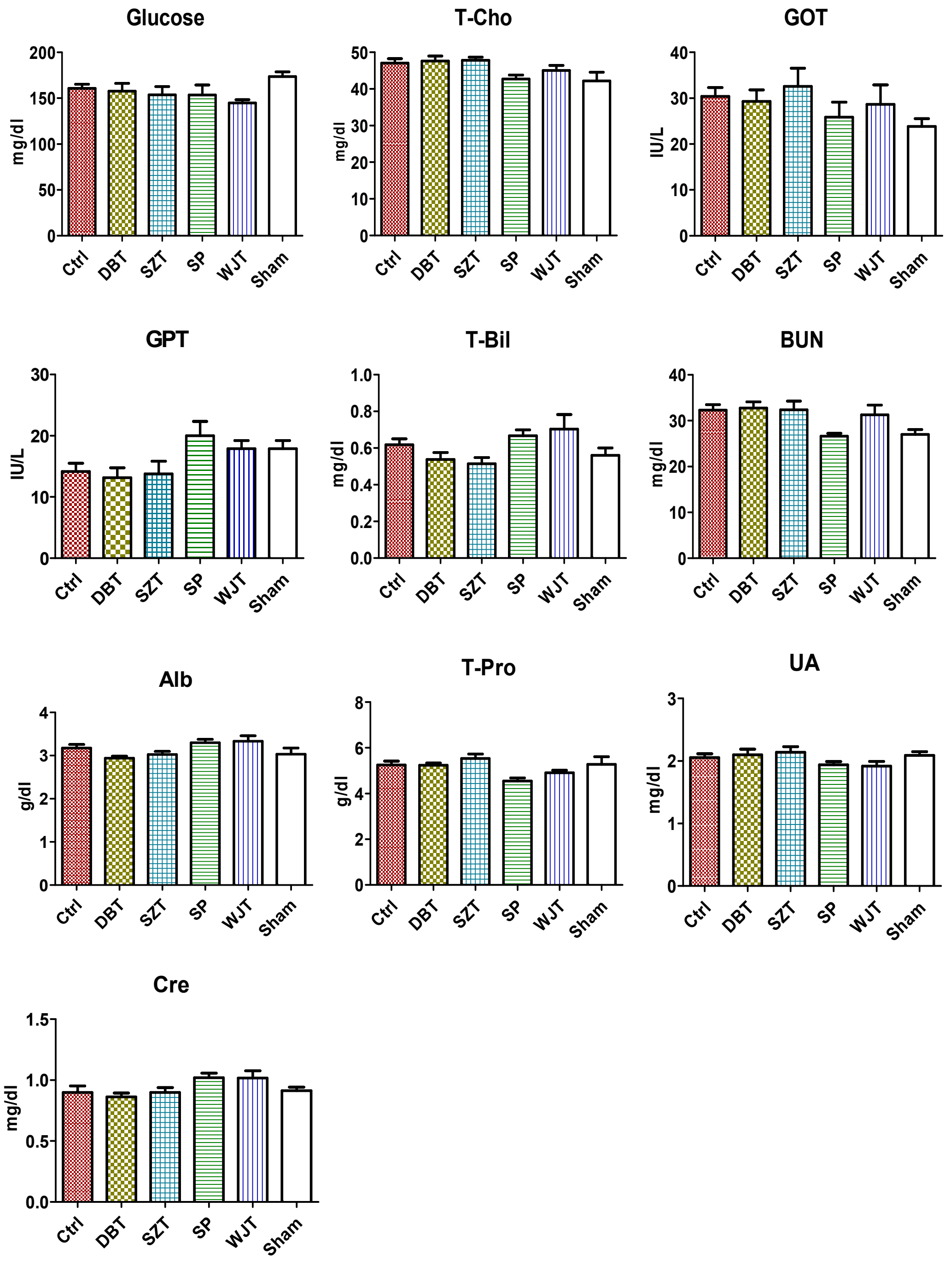

Figure S3. Biochemical tests for health check in TCHM-treated mice. To consider the possible side effects of TCHM treatments on liver and kidney, blood samples were collected on Day 47 (Fig. 1A) and subjected to regular biochemical tests. Those tests include those for metabolism: blood glucose (glucose), total cholesterol (T-Cho); liver function: glutamate oxaloactate transaminase (GOT), glutamate pyruvate transaminase (GPT), bilirubin blood test (T-Bil); renal function: urea nitrogen in the blood (BUN), albumin (Alb), total protein (T-Pro), uric acid (UA), creatinine (Cre). Our studies confirmed no obvious toxicity on liver and kidney after six-week treatments of TCHM in the treated mice.

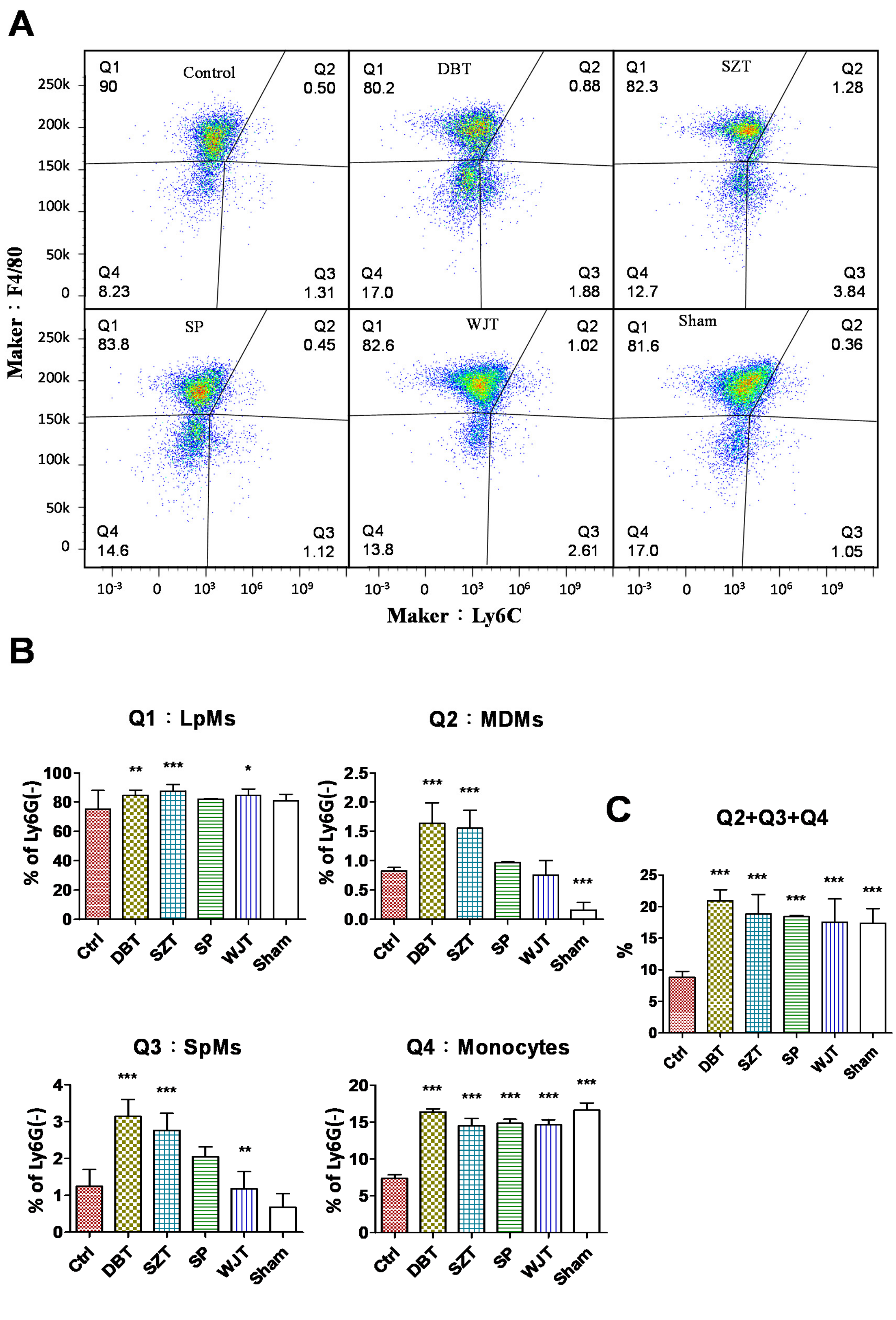

Figure S4. Recruitment of circulating monocytes by TCHMs in the peritoneal cavity of mice with induced endometriosis. (A) Multicolor flow cytometry was applied to analyze the origins of monocytes in the peritoneal cavity of TCHM-treated mice with induced endometriosis by

using the gating strategy shown in Fig. S1. The origins of peritoneal monocytes can be divided into four categories (Q1 to Q4) based on the expression levels of F4/80 and Ly6C. The depicted cellular gating is representative of individual treatments. (B) The bar charts summarized the calculated amounts of LpMs (Q1), monocyte-derived macrophages (MDMs) (Q2), SpMs (Q3), and regular monocytes (Q4). (C) The bar chart summarized the sum of monocytes recruited from the circulation system (Q2+Q3+Q4). Statistical differences between TCHM-treated mice and untreated controls were compared by using t-test. Sham healthy mice served as the negative controls. The p-values were presented as *: p-value < 0.05, **: p-value < 0.01, and ***: p-value < 0.001.
