# Supplementary material for: Traditional Chinese herbal medicines suppress endometriosis development through modulating macrophage-mediated immune responses in the peritoneal cavity
Source: Biomedicine (Taipei). 2026 Jun 1;16(2):35–51. doi: 10.37796/2211-8039.1706 (PMC13387404; doi:10.37796/2211-8039.1706)
Supplement: Supplementary file 2 [file bmed-16-02-035-s002.docx]

**Table S1.** Compositions and daily dosages of traditional Chinese herbal medicine (TCHM) formulae used in this study^a^

| **TCHM (Cat. No./batch No.)** | **Human dosage^b^** | **Composition & Preparation^c^** | **Mouse dosage^d,e^** |
| --- | --- | --- | --- |
| **DBT:** Dang-Qui Bu-Xue Tang (BTI: 3570 / M20026) | 6.0 g per day | The 6.0g powder is prepared from the ointment generated by following dried herbs  dried root of *Angelica sinensis* (Oliv.) Diels 4.0 g  *Astragalus membranaceus* Moench 20.0 g | (6000 mg/60 kg) *12.3*(20/1000) kg = 24.6 mg per day |
| **SZT:** Shao-Fu Zhu-Yu Tang (STPC: 0415 / 19112535) | 12.0 g per day | The 12.0 g powder is prepared from the ointment generated by following dried herbs  dried fruit of *Foeniculum vulgare* (Mill.) Holub 0.2 g  dried root of *Zingiber officinale* Roscoe 0.4 g  *Corydalis yanhusuo* (Y.H.Chou & Chun C.Hsu) W.T.Wang ex Z.Y.Su & C.Y.Wu 2.0 g  *Commiphora myrrha* (T.Nees) Engl. 2.0 g  dried root of *Angelica sinensis* (Oliv.) Diels 6.0 g  *Conioselinum anthriscoides* (H.Boissieu) Pimenov & Kljuykov 2.0 g  *Cinnamomum cassia* (L.) D.Don 2.0 g  dried root of *Paeonia lactiflora* Pall. 4.0 g  dried pollen of *Typha angustifolia* L. 6.0 g  dried stool of *Trogopterus xanthipes* Milne-Edwards 4.0 g | (12000 mg/60 kg) *12.3*(20/1000) kg = 49.2 mg per day |
| **SP:** Shi-Xiao Powder  (SPTC: 5423 / 18080301 for Wu-Ling-Zhi; SPTC: 6410 /18080304 for Pu-Huang) | 6.0 g per day | The 6.0g powder is prepared from the ointment generated by following dried herbs  dried stool of *Trogopterus xanthipes Milne-Edwards* (Wu-Ling-Zhi) 15.5 g  dried pollen of *Typha angustifolia* L. (Pu-Huang) 2.5 g | (6000 mg/60 kg) *12.3*(20/1000) kg = 24.6 mg per day |
| **WJT:** Wen Jing Tang  (STPC: 1302H / 18053133) | 13.5 g per day | The 13.5 g powder is prepared from the ointment generated by following dried herbs  dried fruit of *Tetradium ruticarpum* (A.Juss.) T.G.Hartley 3.0 g  dried root of *Angelica sinensis* (Oliv.) Diels 2.0 g  *Conioselinum anthriscoides* (H.Boissieu) Pimenov & Kljuykov 2.0 g  dried root of *Paeonia lactiflora* Pall. 2.0 g  dried root of *Panax ginseng f. dolichocarpus* Makino 2.0 g  *Cinnamomum cassia* (L.) J.Presl 2.0 g  dried skin of *Equus asinus* L. 2.0 g  dried root skin of *Paeonia × suffruticosa* Andrews 2.0 g  fresh root of *Zingiber officinale* Roscoe 2.0 g  *Glycyrrhiza glabra var. uralensis* (Fisch. ex DC.) L.Duan 2.0 g  *Pinellia ternata* (Thunb.) Makino 3.0 g  *dried root of Ophiopogon japonicus* (Thunb.) Ker Gawl. 4.0 g | (13500 mg/60 kg) *12.3*(20/1000) kg = 55.4 mg per day |

^a^Those TCHM formulae were purchased from Sun Ten Pharmaceutical Co. Ltd. (STPC), Taipei, Taiwan or Biotanico Inc. (BTI), Tainan, Taiwan. These two are GMP-certified pharmaceutical companies which are considered as key TCHM providers around the world.

^b^The suggested dosage by the pharmaceutical companies.

^c^ The plant names have been checked with World Flora Online (www.worldfloraonline.org).

^d^The mouse dosage was calculated from the human dosage based on the guidance of Food and Drug Administration (FDA)/Center for Drug Evaluation and Research (CDER) in USA (Nair et al., 2016). Under such regulation, an adult is considered as 60.0 kg and a mouse is considered as 20.0 g. The correction factor (Km) of the dosage from human to mouse is 12.3.

^e^Corn starch was used as the pre-gelatinized starch in the preparation of the TCHMs. Thus, the untreated control mice in this study were fed with corn starch (24.6 mg per day per mouse).
