# Supplementary material for: Traditional Chinese herbal medicines suppress endometriosis development through modulating macrophage-mediated immune responses in the peritoneal cavity
Source: Biomedicine (Taipei). 2026 Jun 1;16(2):35–51. doi: 10.37796/2211-8039.1706 (PMC13387404; doi:10.37796/2211-8039.1706)
Supplement: Supplementary file 3 [file bmed-16-02-035-s003.docx]

**Table S2.** The antibodies utilized for flow cytometry study

| **Antigen** | **Format** | **Cell specificity** | **Dilution factor** | **Manufacturer** | **Catalog No.** |
| --- | --- | --- | --- | --- | --- |
| F4/80 | PE-Cy7 | Macrophage | 1/200 | Biolegend | 123114 |
| Ly6C | PE | Monocytes | 1/400 | BD | 560592 |
| MHC-II | APC-Cy7 | SpM | 1/400 | Biolegend | 107627 |
| Ly6G | PE-Texas Red | Neutrophils | 1/200 | BD | 562700 |
| CD45.2 | PerCP-Cy5.5 | Leukocyte | 1/200 | Biolegend | 109827 |
| CD3 | PE/Dazzle | T-cell | 1/80 | Biolegend | 100347 |
| CD124 | PE | Th1 | 1/200 | Biolegend | 144803 |
| CD11c | APC-Cy7 | Macrophage-M1 | 1/200 | Biolegend | 117323 |
| IFN-γ | PerCP-Cy5.5 | Th2 | 1/200 | Biolegend | 505821 |
| CD4 | FITC | Th cell | 1/400 | BD | 553650 |
| CD206 | BV421 | Macrophage-M2 | 1/400 | Biolegend | 141717 |
| CD19 | APC | B cell | 1/200 | Biolegend | 152410 |
| CD49b | APC | NK cell | 1/200 | BD Pharmingen | 560628 |
| Siglec F | APC | Eosinophils | 1/200 | Miltenyi | 130-102-241 |
| FVS660 | APC | Dead cell | 1/1000 | BD | 564405 |

| **Antigen** | **Conjugate** | **Antibody type** | **Dilution factor** | **Manufacturer** | **Catalog No.** |
| --- | --- | --- | --- | --- | --- |
| Antibodies for IHC study | | | | | |
| Vimentin |  | Primary antibody | 1/50 | Cell | 5741 |
| Cytokeratin |  | Primary antibody | 1/50 | Abcam | Ab93279 |
| Polymer Detection Systems | HRP | Secondary antibodies | 1/1 | Novolink | RE7140-CE |
| Goat anti Mouse IgG | HRP | Secondary antibody | 1/500 | Arigo | ARG65350 |
| Antibodies for IF study | | | | | |
| PGP9.5 |  | Primary antibody | 1/100 | Millipore | AB1761-I |
| CD31 |  | Primary antibody | 1/2000 | Novus | NB100-1642 |
| LYVE-1 |  | Primary antibody | 1/100 | GeneTex | GTX53124 |
| ICAM-1 |  | Primary antibody | 1/250 | Invitrogen | MA5407 |
| AKAP12 |  | Primary antibody | 1/200 | Protentech | 125199-1-AP |
| Goat anti Mouse IgG | CFL555 | Secondary antibody | 1/2000 | Santa Cruz | Sc-362267 |
| Goat anti Rabbit IgG | Alexa Fluor-488 | Secondary antibody | 1/500 | Jackson | 111-545-144 |
| Donkey anti Rat IgG | Alexa Fluor-594 | Secondary antibody | 1/500 | Jackson | 712-585-153 |
| Goat anti Rabbit IgG | Rodamine | Secondary antibody | 1/1000 | ABgent | ASR1424 |
| Goat anti Rat IgG | FITC | Secondary antibody | 1/500 | Arigo | ARG23755 |

**Table S3.** The antibodies utilized for immunohistochemistry (IHC) and immunofluorescence (IF) staining

**Table S4.** Sequences of primers utilized for quantitative PCR study

| IL-6 | F | 5’-catgttctctgggaaatcgtgg-3’ |
| --- | --- | --- |
|  | R | 5’-ccactccttctgtgactccagc-3’ |
| IL-1β | F | 5’-gagaatgacctgttctttgaag-3’ |
|  | R | 5’-cgttgcttggttctccttgtac-3’ |
| IGF-1 | F | 5’-ccttccaactcaattatttaag-3’ |
|  | R | 5’-ggagccatagcctgtgggcttg-3’ |
| TNF-α | F | 5’-caaagggatgagaagttcccaaatgg-3’ |
|  | R | 5’-cactccagctgctcctccacttggtg-3’ |
| COX-2 | F | 5’-ctcatactcataggagagactatc-3’ |
|  | R | 5’-cagcaacccggccagcaatc-3’ |
| GAPDH | F | 5’-catcactgccacccagaagactg-3’ |
|  | R | 5’-atgccagtgagcttcccgttcag-3’ |
